# Pigmented squamous cell carcinoma in a non-photo-exposed area of an indigenous woman^☆^

**DOI:** 10.1016/j.abd.2022.06.008

**Published:** 2023-09-23

**Authors:** Luana Amaral de Moura, Lucia Martins Diniz, Emilly Neves Souza, Lucas Amaral de Moura

**Affiliations:** a Department of Dermatology, Hospital Universitário Cassiano Antônio Moraes, Universidade Federal do Espírito Santo, Vitória, ES, Brazil; b Faculdade Multivix, Cachoeiro de Itapemirim, Itapemirim, ES, Brazil

Dear Editor,

A 67-year-old indigenous woman, living in a reservation in the north of the state of Espírito Santo, Brazil, previously hypertensive and a smoker, reported an erythematous area on her left thigh of more than ten years duration, with radial growth and mild pruritus. On examination, she had an infiltrated erythematous, brownish, hyperkeratotic plaque on the proximal portion of the left thigh, a non photo-exposed area (Fig. 1). There was no evidence of solar elastosis around the lesion. Inguinal lymph node enlargement was not identified. Dermoscopy showed deposits of a black pigment, erythema, and central linear vessels, in addition to glomerular vessels and peripheral striae.

**Figure 1:**
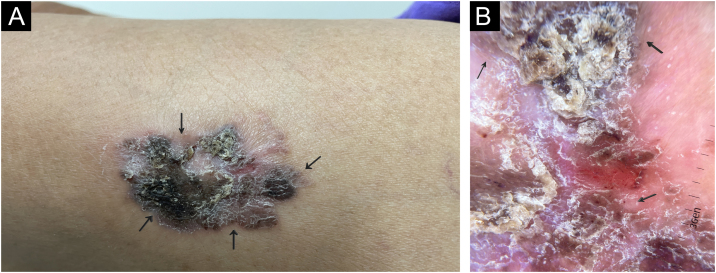
(A) On examination, an infiltrated erythematous-brownish plaque was observed, surmounted by a hyperkeratotic area, in the proximal lateral region of the left thigh (covered area). (B) Dermoscopy showing erythema and linear vessels in the central region, areas of black pigment and glomerular vessels and radiated pigment in the periphery.

The main diagnostic hypotheses were Bowen's disease, melanoma and verrucous syndrome (PLECT - paracoccidioidomycosis, leishmaniasis, sporotrichosis, chromomycosis, cutaneous tuberculosis).

Cultures were performed for fungi and bacteria, which were negative. Histopathological evaluation of an incisional biopsy showed compact hyperkeratosis, acanthosis, impaired cell maturation, pigment deposits, without an increase in melanocytes, in addition to atypical keratinocytes and mitoses in the middle portion of the epidermis, confirming pigmented Bowen's disease (Fig. 2).

**Figure 2:**
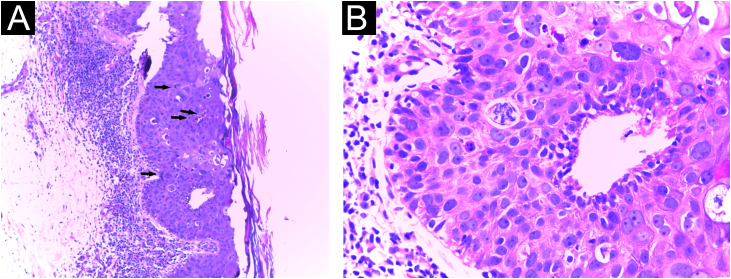
Histopathology of an incisional biopsy, which suggested the diagnosis of pigmented SCC *in situ*. (A) Compact hyperkeratosis, acanthosis and pigment deposits, in addition to atypical keratinocytes and mitoses in the middle portion of the epidermis (Hematoxylin & eosin, ×40). (B) At higher magnification, atypical keratinocytes and mitoses (Hematoxylin & eosin, ×400).

Initially, imiquimod 50 mg/g cream was prescribed to reduce the lesion and facilitate excision, but without a satisfactory response. The lesion was excised with a Limberg flap, and the anatomopathological analysis showed invasion of the deep reticular dermis, characterizing pigmented squamous cell carcinoma (SCC - Fig. 3). Immunohistochemistry showed positivity of keratinocytes for EMA (epithelial membrane antigen) and p53 and p63 proteins, confirming the diagnosis (Fig. 4).

**Figure 3:**
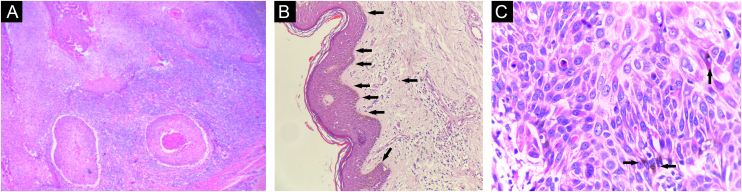
Histopathology of the excisional biopsy. (A) Invasive cutaneous neoplasm consisting of atypical and pleomorphic cells with clear eosinophilic cytoplasm and pleomorphic nuclei with abundant keratinization (Hematoxylin & eosin, ×100). (B) Pigment deposits (Hematoxylin & eosin, ×40). (C) At higher magnification, keratinocyte atypia and pigment deposits (Hematoxylin & eosin, ×400).

**Figure 4:**
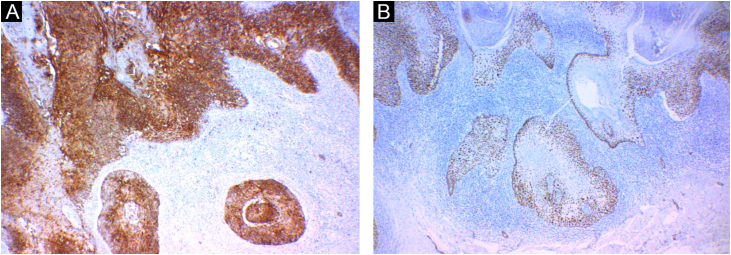
Immunohistochemistry. (A) Positivity for EMA (epithelial membrane antigen). (B) Positivity for p63 protein.

Squamous cell carcinoma (SCC) accounts for 20% to 50% of skin cancers in Brazil. It is more common in Caucasians and in those over 60 years of age, with exposure to ultraviolet radiation being the main risk factor.^1^ One of its variants is the pigmented SCC, a rare and poorly described subtype.^2^

Pigmented SCCs represent between 0.01% to 7% of all SCCs according to the literature in English, although other authors have identified a prevalence of almost 25% (due to the inclusion of tumors with only small areas of pigmentation).^2^, ^3^

The first Brazilian case of this subtype was published in 2009, describing a blackish exophytic nodular lesion in the right malar region, with a one-year evolution, in an elderly Caucasian woman.^4^ This is considered the typical manifestation of pigmented SCC: rapidly evolving pigmented papule or plaque in a photoexposed area (especially head and neck), in elderly patients.^5^ The literature shows only one case of a lesion in a non-photo exposed area, such as the present report, which was a brownish nodule with ulceration in the right lumbar region.^5^

There is no specific dermoscopic description for pigmented SCC. The few publications used terms that are characteristic of melanocytic lesions, such as striae, globules, and homogeneous blue pigmentation, or of keratinized tumors, such as atypical vessels and whitish halo associated with the keratinization process.^3^, ^6^

The differential diagnosis is challenging and includes melanoacanthoma, melanoma, and squamomelanocytic tumor, as well as pigmented variants of basal cell carcinoma, Bowen's disease, actinic keratosis, and pilomatricoma.^5^ Therefore, histopathology is essential for diagnostic confirmation, which may reveal proliferation of atypical keratinocytes and frequent mitoses. The pigment can be seen both in the cytoplasm of keratinocytes and in dispersed, non-neoplastic melanocytes and melanophages in the surrounding stroma.^2^

Immunohistochemistry can help in the diagnosis. Epithelioid cells express EMA (epithelial membrane antigen), p40 and p53 proteins, and high and/or low molecular weight cytokeratins. Melanocytes may exhibit positive immunoreactions for S-100, tyrosinase, and HMB-45.^2^, ^4^, ^7^ HMB-45 is a mouse monoclonal antibody against Pmel17/gp100, thought to be specific for activated or neoplastic melanocytes, such as melanoma cells. In HMB-45-positive cases of pigmented SCC, the expression of this marker is thought to be secondary to melanocyte stimulation in response to antigens released by the tumor.^5^

Several terms are used in an attempt to characterize tumors consisting of two or more types of neoplastic cells. Pigmented SCC seems to be part of those described as colonized. In these, a group of tumor cells is deposited around and colonizes another pre-existing neoplasm. This behavior can be observed in benign or malignant epithelial neoplasms that are secondarily populated by melanocytes without atypia, such as pigmented actinic keratosis, melanoacanthoma, and pigmented basal cell carcinoma. The dynamics involved in the melanocytic colonization of these tumors have yet to be elucidated.^5^, ^7^

The biological behavior of this neoplasm is unclear due to the limited number of publications, but its course is believed to be similar to that of conventional SCC.^5^ The present report emphasizes the importance of including pigmented SCC as a differential diagnosis of pigmented lesions, even in atypical locations and in non-Caucasian patients.

## Financial support

None declared.

## Authors' contributions

Luana Amaral de Moura: Design and planning of the study; drafting and editing of the manuscript; collection, analysis, and interpretation of data; critical review of the literature; critical review of the manuscript.

Lucia Martins Diniz: Design and planning of the study; effective participation in research orientation; intellectual participation in the propaedeutic and/or therapeutic conduct of the studied case; critical review of the literature; critical review of the manuscript; approval of the final version of the manuscript.

Emilly Neves Souza: Design and planning of the study; drafting and editing of the manuscript; collection, analysis, and interpretation of data; critical review of the literature.

Lucas Amaral de Moura: Design and planning of the study; drafting and editing of the manuscript; collection, analysis, and interpretation of data; critical review of the literature.

## Conflicts of interest

None declared.
